# Erwin Deutsch, the Eppinger Clinic and the legacy of the Second Vienna School of Medicine—Continuities of a career

**DOI:** 10.1007/s00508-022-02045-8

**Published:** 2022-06-13

**Authors:** Clemens Jobst, Herwig Czech

**Affiliations:** Ethics, Collections and History of Medicine (Josephinum), Medical University of Vienna, Währinger Straße 25, 1090 Vienna, Austria

**Keywords:** History of (internal) medicine, Vienna Medical School, Internal medicine, National Socialism, Austrian postwar history

## Abstract

Erwin Deutsch (1917–1992) was an outstanding representative of Austrian internal medicine after World War II. Little is known about his early biography. Considered a “Jewish half-breed” under Nazi racial laws, he was subjected to harassment during his training. Nevertheless, he can be regarded as scientific heir of Hans Eppinger (1879–1946), who enjoyed a worldwide reputation as internist despite his controversial involvement in medical experiments in the Dachau concentration camp.

Already declining after World War I, the Viennese Medical Faculty largely lost its international scientific importance with the expulsion of over half its faculty members from 1938, the end of the Second Vienna School of Medicine. Erwin Deutsch significantly contributed to continuity by vehemently calling for the unity of internal medicine after 1945, as it had been practiced in Vienna since the nineteenth century. Discrimination as a “Jewish half-breed” played a paradoxical role in this context—it delayed the start of his independent academic activity and increased his personal dependence on Eppinger; at the same time it spared him military service and enabled him to start his career after 1945 unaffected by denazification measures.

Based on unpublished archival material, interviews with contemporary witnesses, and Deutsch’s medical publications, this article is the first to offer an account of his early career, from his graduation in 1940, his time at the Eppinger Clinic, compulsory service in Germany during the war and the beginning of his scientific work to his appointment as Ernst Lauda’s successor as director of the 1st Medical Clinic in Vienna.

## Introduction

Proposing an appropriate candidate to head the 1st Medical Clinic in Vienna (*1. Medizinische Universitätsklinik*) posed a difficult task for the appointment committee in 1964. The eight signing professors reminisced about the past glory days of the renowned (Second) Vienna School of Medicine (*Zweite Wiener Medizinische Schule*), which needed a candidate to step into the shoes of the predecessors Josef Škoda, Hermann Nothnagel, Karel Frederik Wenckebach, Hans Eppinger jun., and most recently Ernst Lauda. According to the committee, Lauda had “died too early,” while the following generation of clinicians was still gaining a foothold after a 10-year hiatus in scientific life in the Viennese medical faculty from 1940 to 1950. Nevertheless, they produced candidates with impressive scientific résumés, of whom Erwin Deutsch, shortlisted in primo loco before two colleagues, ultimately succeeded.^1^

But why the reluctant tone? Was it regret that they could not produce a candidate who had intimate ties to the academic elite like his predecessor [1]?^2^ Was it nostalgia for the golden days of the Vienna School of Medicine, which had slowly bled out and died due to an unprecedented brain drain in the 1930s, peaking in 1938 [2]? Were there anti-Semitic preconceptions against the candidate in primo loco, who had been labelled a half-Jew in Nazi Austria and as such had to endure various reprisals? To what extent had the sciences at the clinic during Nazism indeed been “mostly dead,” as seems to have been the view in the 1960s?^3^

The following article tries to answer these questions via a biographical approach, following Erwin Deutsch’s career at the 1st Medical Clinic. Deutsch was born in 1917 in Klagenfurt (Carinthia) into a middle-class, Bohemian-Viennese family. When he started his studies of medicine in 1935, his father had already passed away. Julius Deutsch had been an official for the Imperial-Royal State Railways (as *Zentralinspektor*). Despite having been baptized himself in 1890, his son Erwin was classified, according to the Nuremberg Laws, as *Mischling 1. Grades* (“half-Jew”) after the 1938 annexation (*Anschluss*), due to his descent from two Jewish grandparents. As for many others in a similar situation, this meant that his matriculation could be revoked at any time.

## *Mischlinge* in medicine under the Nazi regime

Following the *Anschluss*, about 2200 students who, according to the Nuremberg Laws were considered Jewish, were expelled from the University of Vienna. The situation for *Mischlinge* (people of mixed Jewish and non-Jewish descent) was less clear. Some, like Deutsch, were allowed to graduate under reservation of revocation^4^ but were denied the license to practice. Younger semesters had to apply at the dean’s office for permission to study due to a legal amendment in 1940, which aimed to get rid of *Mischlinge *of the first degree (“half-Jews”) and to assimilate *Mischlinge *of the second degree (“quarter-Jews”). Indeed, exemptions were made, and applicants that “barely looked Semitic” and wrote their pleas convincingly enough had chances to obtain their MD, but without being allowed to practice [3].^5^

Many tried to hide or simply had no awareness of their Jewish ancestry. Some of them even were Nazis like the neurologist Walther Birkmayer, co-discoverer of levodopa effects for the treatment of Parkinson’s disease. As an early member of the SS and the Nazi party, when it was illegal in Austria, he contributed to the expulsion of Jewish personnel from the Vienna Medical Faculty. Upon being exposed as a *Mischling*, Birkmayer was stripped of his Nazi functions but could retain his professional status for the remainder of the war with support from leading Viennese Nazis and continued his career unimpeded thereafter [4]. Others were not so lucky in being part of a safeguarding network: Professor Gustav Bayer, student of Sigmund Exner, internationally renowned endocrinologist and *Mischling*, was expelled from the Medical Faculty of Innsbruck. Fearing persecution, he and his 17-year-old daughter took their own lives 2 days after the *Anschluss *[5].

*Mischlinge*, like Jews, were to be dismissed from public hospitals, even though some were allowed to stay and work without payment [6]. There is evidence of some (even SS-affiliated) leading personnel protecting their co-workers and providing them with relatively safe workspaces [1, 7].^6^ Deutsch entered the 1st Medical Clinic in 1940 as a guest doctor, meaning without salary. Despite endeavors of his superiors who tried to keep him in Vienna, Deutsch was assigned to serve in various German clinics during the following years.^7^

Regarding the treatment of *Mischlinge*, the regime had no consistent policy. *Mischlinge *of the first degree (“half-Jews”) were discussed to be subjected to mass sterilization, or to be murdered alongside the Jewish population in general. Although this ultimately did not come to pass, their fate varied depending on their marital status, children, religious denomination, and other factors. During the war, they were in constant danger of being deported for minor infractions [8]. Some were coerced into the labor battalion *Organisation Todt* to work in large construction projects such as the Siegfried Line (*Westwall*). The application of the Nuremberg Race Laws to Wehrmacht soldiers in 1940 theoretically meant an exclusion of *Mischlinge*. Until then, many had volunteered for service in hopes of being rewarded with a German Blood Certificate (*Deutschblütigkeitserklärung*) by Hitler himself for deeds at the front. Additionally, the camaraderie among the troops contributed to a sense of identity for previously excluded members of society. In practice, many *Mischlinge* were not discharged, be it through hiding their racial status or through protection from superiors. During the course of the war, personnel shortage not only in the Wehrmacht but also in various other organizations grew, thus allowing some *Mischlinge* to occupy positions befitting their expertise. Generally, racist measures against *Mischlinge *escalated as the war progressed. On the other hand, lack of personnel in many areas opened loopholes and opportunities for a measure of protection [9, pp. 230–251].

The repressions against Erwin Deutsch included the aforementioned coerced transfers, ban from patient contact during a temporary return to Vienna, and scientific silencing, hampering his efforts to share important research on diuretics and kidney physiology [10, 11].^8^ His application for approbation was ultimately granted in 1943 from Berlin, thus making patient care possible again.^9^ Despite discrimination, Deutsch seemingly never directly fought his oppression and rather aimed to blend in. It is even possible that he sympathized with some aspects of National Socialism: According to a 1942 political assessment in his NSDAP personnel file (*Gau-Akt*), he had associated with illegal Nazi circles during the Austrofascist regime.^10^ The reliability of this document is however questionable, since it falsely ascribed Jewish faith to Deutsch and labelled him as a military doctor.^11^

## Inhumane experiments

The 1st Medical Clinic is notorious for its involvement in experiments on Dachau concentration camp prisoners. Department head Eppinger lobbied for a Viennese method of making seawater drinkable. Via his assistant and “most capable student,”^12^ SA member Wilhelm Beiglböck, Eppinger was involved in human experiments on Roma and Sinti and personally visited Dachau at least twice [12 p. 147]. Most of the test subjects had survived Auschwitz, where the gypsy camp was liquidated in August 1944. After the liberation, a former Dachau detainee recognized Beiglböck in a British detention camp and informed authorities that this was the doctor who had performed coerced experiments and was responsible for the deaths of two Romani in autumn 1944. Another witness reported the concealment of a fatality by moving the test subject back to his regular quarters. The actual death toll, as well as the exact methodological design of the Dachau seawater experiments, are to this day subject of debate [12 pp. 133–135]. Between 40 and 60 people were used as test subjects, divided into four groups and subjected to different experimental regimens. Soon suffering from convulsions, extreme thirst and delirium, some managed to sneak out of their rooms or to suckle washing water out of cleaning rags [12, pp. 146–157].

Beiglböck was the only Austrian doctor accused in the post-war Nuremberg doctors’ trial [12 pp. 138–139, 7, p. 53].^13^ When Eppinger was called to testify, he committed suicide.^14^ Whether the call to testify was indeed the motive for his suicide is subject of debate. Erwin Deutsch, while acknowledging the Dachau experiments, denied Eppinger’s role in using inmates as test subjects. Having explicitly stated his wish for a personal rehabilitation of Hans Eppinger to historian Erna Lesky,^15^ Deutsch claimed in an apologetic obituary that the true reasons for his mentor’s suicide were the loss of close relatives in the war and the inability to accept the obsolescence of certain scientific hypotheses [13].

Years before the discovery of hepatitis viruses, Eppinger’s theory of “serous inflammation” regarded jaundice as the result of malnutrition or intoxication. To prove this theory, his assistant Lainer conducted the *experimentum crucis* and transfused blood and duodenal juice from icterus patients to 15 healthy patients—with no mention of consent [14, 15 p. 139]. When they did not develop symptoms within 14 days, Lainer concluded that jaundice was indeed not infectious. There was no further surveillance of the subjects, meaning that likely liver damage resulting from the experiments would remain undetected [7, p. 48].

Further ethical transgressions include a cooperation with Otto Pötzl’s Psychiatric-Neurological Clinic, where Eppinger supervised a doctoral dissertation on insulin shock and salt metabolism (a topic also relevant to the Dachau seawater experiments). Without therapeutic motivation, artificial hypoglycemia was induced by administering insulin shocks—an agonizing process, only relieved by hypoglycemic coma [16, 17 p. 638]. Similar human experiments were conducted for another dissertation investigating vitamin C metabolism under insulin shock [18, 17 p. 638]. Eppinger and Pötzl had instructed Beiglböck to explore metabolic processes under insulin shock as early as 1936. After systematic clinical trials on 80 patients, he concluded that digestive ailments ameliorated after “non-therapeutically induced hypoglycemia” [19, 17, p. 638].

Little is known about experiments in the 1st Medical Clinic’s low-pressure chambers apart from a connection to the death of alleged serial killer Bruno Lüdke, who was studied as the prototypical “born criminal” and might have been killed in oxygenation experiments.^16^ The pressure chamber (it was 1 among 20 in the German Reich) was constructed before the war to be used for “military purposes” by the Luftwaffe and dismantled after a fire in 1944. Clinic and Luftwaffe were granted joint use of the chamber and received a research assignment (contents unknown) by *Reichsminister* of Aviation Hermann Göring [21]. This chamber for aviation medicine studies at the clinic was no secret to aspiring personnel: Erwin Deutsch knew of its existence and claimed that people had died in there. “You can still see the pipes,” he told an assistant when asked about coerced research in 1992.^17^

While experimental treatment via transplantation of calf pituitaries was performed and yielded promising results in patients with diabetes insipidus, rumors among Viennese physicians connected Eppinger’s suicide to alleged transplanted pituitaries of execution victims to patients suffering from emaciation [22]. Ludwig Popper claimed that the 1st Medical Clinic’s assistant Dietrich Roller (1909–2001) had murdered patients via intracardial phenol injections, a killing method of choice of concentration camp doctors.^18^ Evidence of further possible experiments at the clinic is provided by French military intelligence, which documented reports from an informant that Eppinger had experimented on patients with heart conditions by artificially inducing increased heart rates, in two cases with lethal results [15].^19^

## Eppinger—“highly gifted, yet insane”?

Adept in all facets of internal medicine, Eppinger’s specialty was hepatology. Among his patients were personalities like King Boris of Bulgaria, Josef Stalin and Kemal Atatürk, indicating his international prestige. His character, however, was debatable: “Although highly gifted, he was an insane man,” his colleague Sigismund Peller stated after having witnessed Eppinger opening a patient’s arteria radialis without medical indication [24, p. 81]. In his inauguration speech, Eppinger invoked the medical ethos of nihil nocere [25, p. 772]. Several testimonies about his practices, however, paint a different picture. There are reports that he performed liver biopsies without patient consent or therapeutic indications. Allegedly, he also directed his assistant Hans Popper to distract the clinic head K. F. Wenckebach in order to steal testes from a shipment of torsi from Indonesia for the study of beriberi. Eppinger was interested in edematous phenomena and used the stolen tissue to test his hypothesis of “serous inflammation.” He was the first professor from the Viennese faculty to receive a ban from the College of Physicians in Vienna (*Gesellschaft der Ärzte in Wien*) library after cutting out various articles for personal use, and he dissected his own daughter after she had died of diphtheria [7, pp. 41–46]. Multiple charges due to speeding,^20^ spitting down on patients from the elevator or urinating in hospital sinks or into rivals’ offices [26, p. 238] are other reported examples of behavior ranging from the eccentric to the offensive.

Still, it seems that personnel of Eppinger’s clinic had nothing but admiration for him [13, 7 p. 26, 26]. From a political standpoint, he was attributed a kind of *naïveté* when he believed that the Nazi intermezzo would only last for a short while [7, p. 46]. Despite having entered the Nazi party in 1937, effectively joining a terrorist organization (the NSDAP was banned by the Austrofascist regime 1933–1938), Eppinger was not regarded as an enthusiastic proponent of the NSDAP by the Nazi party itself, being called, among other things, “everything but a real National Socialist”, “the most despicable character at the medical faculty”, “slanderous, scheming, and relentless.”^21^ When he joined the illegal NSDAP in 1937, he had already been a long-standing member of the pan-German *Deutscher Klub*, a network of right-wing academics who aimed for the Anschluss of Austria to Germany [27].^22^ Some of his contemporaries regarded Eppinger mainly as an opportunist—expressed in the nickname *Je-nachdem-okrat*^23^ [26 p. 237]. Due to his choice of personnel at his previous position in Freiburg and from 1933 onward in Vienna, rumors circulated about Eppinger being Jewish-friendly, of “Jewish appearance” or even part Jewish [28, 7, p. 40].^24^ Even though he stated his sympathies for the Nazi regime in press articles [29] and warned about the “Jewification” of the Vienna School of Medicine [30], party officials did not quite like his critical opening speech at a congress in 1943^25^ [13] and the fact that he had been called to Moscow on several occasions to treat Josef Stalin, which Eppinger simply denied.^26^ Still, for someone who was despised that much, he was able to hold onto his authority, seemingly unquestioned by his assistant staff, which after the dismissal of the Jewish doctors mainly consisted of SS men, most of which were dismissed in 1945.^27^ Maybe the political assessment concerning Eppinger’s character was exaggerated? Erwin Deutsch, who was discriminated against by the Nazi regime, praised his mentor’s character, especially in his handling of marginalized members of his clinic^28^ [13] as seen in Eppinger’s attempt to oppose Deutsch’s obligatory assignments to German hospitals as well as appreciative letters of recommendation for personnel who were expelled after the *Anschluss*.^29^

Immediately after the end of the war, Deutsch tried in vain to return to Vienna. Since the travel permit he received from the French forces occupying the Saarland was rejected by US forces at the Austrian border, Deutsch had to turn back and only received a valid permit months later.^30^ Stuck in Germany, he was assigned from July to December 1945 to Hans Dietlen’s clinic in Neustadt an der Weinstraße.^31^ This lead to difficulties with accrediting Deutsch’s work in Germany for his training back in Vienna, the rejection of which would have delayed his career even further.^32^ He also was unable to establish contact with his relatives who, unaware of his whereabouts, initiated a missing person inquiry.^33^

Eventually back in Vienna in December 1945, Deutsch rejoined the 1st Medical Clinic in the position of an unpaid auxiliary doctor. In this, he was not the only one: in what was dubbed “slave labor” by a colleague, countless young doctors, many of which were returning prisoners of war, were exploited as scientific workhorses, often unpaid, and could count themselves lucky to be listed as third authors, even if the project was their intellectual property [7, p. 132]. Only a few days after returning to Vienna, however, Deutsch received a coveted paid position thanks to (at the time provisional) clinic head Ernst Lauda.^34^

At this point, Eppinger had already been dismissed as head of the clinic given that he had joined the then illegal NSDAP in 1937 (with a corresponding membership number 6,164,614). Still, he acted as an advisor to the Russian army high command in Austria. After Eppinger’s suicide in 1946, the stigma of an “illegal Nazi” was removed in his denazification procedure by changing the date of his entry to May 1938.^35^ Nowadays, due to the Dachau seawater drinking experiments carried out by his assistant Beiglböck, Eppinger is mostly remembered as an unscrupulous experimenter. Yet, internationally esteemed disciples tried to achieve Eppinger’s rehabilitation. Both Eppinger and Deutsch had a close relationship with Hans Popper, who achieved fame in Chicago as a hepatopathologist in Eppinger’s spirit.^36^ In Vienna, Popper had developed the creatinine clearance test for assessing kidney function, which is still in use today. After the Anschluss, his colleague Falko Lainer (the same who injected patients with icteric blood and duodenal juice [14]) humiliated Popper by locking him in his office overnight. Deciding to flee Vienna, Popper received a fluorescent microscope by Eppinger upon his departure. This facilitated Popper’s career in the USA, as he was able to demonstrate his histological prowess [31]. During the 1980s, Popper—the “father of modern hepatopathology”—was heavily criticized for his fidelity to his mentor Eppinger [32]. While Austria still hesitated to critically assess its Nazi past, an international debate was sparked by Howard Spiro [28], which led to the renaming of the Falk Foundation’s prestigious Hans Eppinger Award to Hans Popper Award in 1990 [33].

## Ernst Lauda and the unity of internal medicine

Although Ernst Lauda was considered by the NSDAP hierarchy as a supporter of the Austrian conservatives, they conceded that he was overall “politically indifferent” and therefore did not object to his unimpeded career during the war [1 p. 165]. After the liberation, good connections and sympathies with the clerical Austrofascist regimeof 1933 to 1938 were characteristic of professorial appointments under dean Leopold Arzt. Lauda, unlike Arzt and Karl Fellinger for instance, largely abstained from expressing political opinions. His actions in this respect seem largely conformist: in the beginning of the Nazi era in Austria, Lauda, together with 12 other conservative university teachers, undersigned a document protesting the proposed emigration of the A.M. A. (American Medical Association of Vienna) to London. This move had been proposed by Dr. J. Landmann due to the dismissal of “a large number of former professors and teachers […] in consequence of their non-Aryan origin.” The signatories denied this, claiming they knew “of not one case of persecution of a professor for his racial or religious adherence” and nonchalantly added the anti-Semitic remark that Landmann was “evidently nursing pathological Jewish hatred” [23 pp. 245–248]. In the wake of the denazification procedures, Lauda lobbied for the restitution of Eduard Pernkopf’s position as head of the Department of Anatomy. NSDAP and SA member Pernkopf was the university’s dean and rector during the Nazi era. Lauda argued that the finalization of Pernkopf’s anatomical atlas should be supported, but his endeavor ultimately was unsuccessful [1 pp. 230–232, 34].^37^ Under the deanships of Karl Lindner (1948/1949) and Ernst Lauda (1949/1950) and historically accompanied by the founding and initial electoral success of the VdU party, which consisted of many former Nazis, 10 university lecturers who had been dismissed in the denazification process were reinstated [23 p. 248].^38^ Lauda, however, opposed the regranting of Erwin Risak’s authorization to teach at a university (*venia legendi*) in 1954, on grounds that the former SS-*Obersturmführer* had implemented the “de-Jewification of the strongly Jewified clinic Eppinger.” While Fellinger endorsed Risak’s plea fearing angry repercussions from *Ehemaligen* circles (former Nazis), Lauda, while conceding the possibility, opposed Risak’s rehabilitation on the grounds of scientific dilettantism and apprehension that Risak might become a role model for young students [1, pp. 114–115].^39^

His aristocratic descent and his medical ethos earned Ernst Lauda the title of “The Last Knight” (of internal medicine). In a time when unambiguous test results via laboratory medicine were placed above everything else, and the continuous emergence and specialization of subdisciplines led to the fear of not being able to see *the whole patient* in a holistic approach, Lauda advocated the concept of “unity of internal medicine”—a concept that his successor Erwin Deutsch tried to follow, but ultimately failed. Still, Deutsch strived to inculcate this holistic concept in the spirit of the Second Vienna School of Medicine in his students.^40^ Following the ethos of renowned Viennese doctors like Joseph Škoda, who developed the holy trinity of diagnostics: anamnesis, auscultation and percussion, the likes of Lauda and Deutsch were skeptical with respect to the segmenting of their discipline. This phenomenon was imported from the USA, the country which took the leading role in medical developments after central European (and especially German-speaking) institutions had been bled dry of most of their best qualified personnel [2]. In a 1982 interview, Deutsch called the establishment of new clinics for cardiology and gastroenterology under Karl Fellinger “villainous […], the biggest mistake […], coup de grâce against the Vienna School of Medicine [35, p. 8].”

In an obituary, Deutsch called Lauda the “last complete internist,” being “probably the last single author” of a three-volume textbook on internal medicine [36, 37]. Despite being conservative in his diagnostic approach, maybe even nostalgically romanticizing the methods of the golden days of the Vienna School of Medicine, Lauda was skeptical but not dismissive of newly emerging and growing medical disciplines like radiology, as well as laboratory medicine and ECG. At the 1st Medical Clinic, Lauda incorporated his research specialty—spleen physiology, hemostasis, endocrinology, in short: all things related to blood—in the form of modern treatment concepts Yes, he was primarily an expert on (differential) diagnostics, which he focused on in his lectures [36]. Supposedly, these lectures were of excellent scientific value but badly frequented since Lauda refused to use microphones and spoke in a low voice. On the same account, Lauda was very economical in the acquisition of expensive devices, sharing his mentor Norbert von Ortner’s disdain for excessive reliance on laboratory diagnostics and radiological imaging. He used a wooden stethoscope, was an introvert, fragile and a morphine user. When Deutsch presented him with an important paper regarding blood coagulation, Lauda’s reaction was: “Why do you even write papers? Just read the textbook” [7, pp. 137–138]^41^. Lauda might sometimes have been wary of Deutsch’s achievements, but when in 1958 he recommended him for an extraordinary professorship, he emphasized Deutsch’s international renown based on the discovery of a new type of hemophilia in 1950.^42^

## Continuities of medical paternalism

When Deutsch succeeded Lauda, he declared Lauda’s aforementioned textbook compulsory literature. This led to surprising continuities between the eras of Eppinger and Deutsch as clinic heads: Under Eppinger, ambitious doctors presented him rare slides of liver biopsies that could support his theory of “serous inflammation” as cause of jaundice by way of connecting the same slides to different patients and forging their inventory numbers [7, p. 48].^43^ Deutsch’s assistants on the other hand redacted laboratory test results of patients that were to be presented in the auditorium, so these would be in line with values given in Lauda’s textbook.^44^ Deutsch held his diligently prepared two-hour long lecture five times per week, anticipating modern teaching concepts by almost always presenting an assistant-chosen patient and discussing anamnesis, status, diagnostic findings and differential diagnostics, proceeding to the general pathology, diagnostics and therapy of the respective diseases. Other than Lauda’s, Deutsch’s lecture, despite being early in the morning for students (7:30 or 8:30), was usually packed [38 p. 53]. Deutsch also continued the authoritarian tradition of clinic leaders as infallible “gods in white.” Whereas today’s paradigm is patient compliance through informed consent, half a century ago patient information was limited to essentials, as to not incite worries with unnecessary ponderings [35, p. 10]. The obligation to wear ties and assistants formally lined up for the ward rounds add to the picture.^45^

One could say that Deutsch’s practices were the culmination of Eppinger’s focus on natural sciences and laboratory medicine and Lauda’s fatherly patient-centered, holistic approach. Eppinger was a fervent experimenter, full of ideas and always looking to improve laboratory methods, regarding patients more like an aggregate of diagnostic results than actual people. Deutsch acquired laboratory experience, forcibly so when he was banned from patient contact due to anti-Jewish discrimination, and later in the clinic’s expanding laboratory workspaces and abroad. Lauda apparently put a lot of trust in Deutsch when he requested the young doctor to handle the clinic’s radiological ward. On the other hand, he had to make do with personnel shortages as consequences of the pre-war expulsion of half of the faculty’s lecturers and professors, compounded by war casualties and, after the liberation, denazification measures. Deutsch claimed that his medical training had been seriously harmed due to being banned from approbation and publishing as well as by coerced transfers.^46^ Nevertheless, during those transfers he had to assume responsible positions in internal medicine and surgery as well as managing a radiological ward.^47^

## Reluctant denazification

Another element of continuity can be seen in the handling of clinic members with a Nazi background, exemplified by the case of Karl-Hermann Spitzy, Deutsch’s second in command. Spitzy had been an illegal NSDAP and SS member since 1934, later even under the SS skull banner.^48^ In 1938, Spitzy and his brother Reinhard guided the Nazi leaders Heinrich Himmler and Ernst Kaltenbrunner on their *Sonnwendreise* (summer solstice tour) through Carinthia and Styria [39]. Nevertheless, his post-war career was largely unimpeded. Thanks to his father-in-law Vinzenz Schumy, co-founder of the Austrian People’s Party, Spitzy underwent a lenient denazification process.^49^

Unlike Erwin Deutsch, Karl-Hermann Spitzy later vehemently denied the occurrence of human experiments at the 1st Medical Clinic.^50^ Under Ernst Lauda, Spitzy assumed leadership of the clinic’s newly founded antibiotics research department and later played a significant role in the development of the acid-proof and therefore orally applicable penicillin V. Having also received acknowledgement beyond specialist circles due to his presentation of the popular 1970s TV show *Die Wiener medizinische Schule heute* (The Vienna School of Medicine Today), Spitzy ascended to professorial ranks when a chair for chemotherapy was founded under Erwin Deutsch. To Deutsch’s likely dismay, the chair was transformed into an independent special clinic for chemotherapy in 1979 [40, 41].

## Against the tide of specialization: the mythical “unity of internal medicine”

While both Lauda and Deutsch as clinicians strived for an all-encompassing knowledge of internal medicine, as researchers they had to focus on a specialty, settling on hematology (or, to be more precise, on the young subspecialty of hemostaseology). This scientific focus was continued through Lauda’s innovative concepts for treating hemophilia patients, and in establishing a specialized coagulation laboratory and a thrombosis service at the 1st Medical Clinic [36]. Deutsch embraced this tradition in his scientific endeavors, which yielded the discovery of a new rare type of hemophilia [42, 43, pp. 167–169]. Through his research, teaching and clinical work, both locally and abroad,^51^ he expanded the 1st Medical Clinic on the groundwork laid by Lauda. In the founding and expanding of institutions like the aforementioned coagulation laboratory, one of the first intensive care units, a dialysis station, a poison information center as well as professorial chairs under the roof of a single internal clinic, he aimed to preserve the unity of internal medicine in the spirit of the Second Vienna School of Medicine. He was also among the critics of the 1975 *Universitätsorganisationsgesetz *(‘University Organization Act’), which he saw as weakening the position of university professors vis à vis students, scientific staff, and the Ministry of Science. As head of the building commission for the new general hospital in Vienna, Deutsch in vain opposed the successive emancipation of independent specialty clinics which had started in 1968 with a cardiological clinic (Fritz Kaindl) and continued in the 1970s with infectiology, gastroenterology, chemotherapy and laboratory medicine [35 p. 8, 38, 44].^52^ In the face of increasing specialization, Deutsch’s rearguard struggle to preserve the “unity of internal medicine” looked increasingly quixotic. An attempt in 1973/1974 to restructure the 2nd Medical Clinic as a cluster of distinct specialty units (“Klinikverband Innere Medizin II”) able to compete with its eternal rival, the 1st Medical Clinic under Karl Fellinger, was shot down by a petition sent to dean Seitelberger by the latter’s medical staff.^53^ It must have been deeply satisfying to Deutsch when, shortly after, he helped thwart his long-time rival Herbert Braunsteiner’s attempt to succeed Fellinger, although he arguably was the strongest candidate.^54^ The price to be paid, however, was a public scandal in which Deutsch was accused of having unduly influenced the process as head of the finding commission [45]. At the end, the successful candidate in 1976 was Georg Geyer, third place on the shortlist sent to the Ministry of Science, who had worked under Deutsch for years and co-authored a compendium of laboratory medicine with him. The decision was ultimately Hertha Firnberg’s, Bruno Kreisky’s minister of Science and Research.^55^ After years of conservative dominance in the academic field [46, p. 124], it is likely that Braunsteiner, who was a co-founder of the Austrian People’s Party (ÖVP), was skipped for political reasons.^56^ Erwin Deutsch, who had begun his medical career under the very real threat of anti-Jewish persecution under the Nazi regime, now found himself at the peak of his career and reputation. Despite his ultimately hopeless attempts to maintain what he considered the necessary “unity of internal medicine,” he embodied more than anybody else the striving after 1945 to restore some of the lost glory of the Vienna School of Medicine.

## Footnotes

1 Austrian State Archives (AT-OeStA), AdR, UWFuK, BMU PA, Sign 21, Deutsch Erwin. Referat über die Besetzung der I. Medizinischen Universitätsklinik in Wien, March 1964. The committee consisted of the professors Böck, Fellinger, Asperger, Brücke, Fuchsig, Hoff, Kunz, Wiedmann. *Secundo* and *tertio loco*, Prof. Hanns Fleischhacker and Prof. Anton Neumayr were proposed.

2 Arias conveys the picture of the post-war professorial council as a “self-contained guild” of aging conservatives, many of whom had enjoyed firm ties to the Austrofascist regime before 1938.

3 “1940–1945 war das wissenschaftliche Leben der Universität weitgehend tot.” AT-OeStA, AdR, UWFuK, BMU PA, Sign 21, PA Erwin Deutsch. J. Böck et al., Referat über die Besetzung der I. Medizinischen Universitätsklinik in Wien, March 1964.

4 “Immatrikulation unter Vorbehalt des Widerrufes”. UAW, Mediziner, W.S. 1938–39. Personal data (*Nationale*) Erwin Deutsch.

5 Vienna University Archive (UAW), MED S 51, “Mischlingsgesuche”. The correspondence between the acting academic instances shows the complexity and ambiguity concerning the handling of *Mischlinge*. About 200 students in the medical faculty were affected.

6 At the 1st Medical Clinic, SS-member Erwin Risak supposedly warned his Jewish colleague Hans Popper that he was about to be arrested in the days following the Anschluss.

7 UAW, 1. Med. Klinik 22. Prof. Hans Eppinger and *Oberarzt* (and SS-member) Dietrich Roller pleaded to authorities to desist from transferring Deutsch.

8 AT-OeStA, AdR, UWFuK, BMU PA, Sign 21, Deutsch Erwin. Co-authorship on two papers concerning kidney physiology and theophylline pharmacokinetics was omitted. Via connections to a Prof. Klare, Bielefeld, Deutsch could however publish two articles in a Swiss journal.

9 AT-OeStA, AdR, UWFuK, BMU PA, Sign 21, Deutsch Erwin.

10 AT-OeStA, AdR, ZNsZ, GA, Gauakt Erwin Deutsch. The file was created when the Vienna NSDAP was asked for a political assessment of Deutsch on the occasion of his application for a license to practice.

11 In reality, Deutsch was baptized as an infant. Evidence of military service could not be procured; however, it is possible that Deutsch’s work in Germany was assessed as such by the responsible authorities.

12 UAW, 1. Med. Klinik 107. Hans Eppinger, reference for Dr. Wilhelm Beiglböck, December 18, 1943.

13 Beiglböck, after several years in prison and failed attempts at professional and academic rehabilitation in Austria, was found dead with a broken neck at the foot of a staircase in his apartment building. The exact circumstances are unknown.

14 Allegedly, Deutsch’s later wife Alma Sitte (1922–1989) in her function of laboratory assistant unwittingly had provided a lethal dose of cyanide to Eppinger, who had claimed to need it for animal experiments. Interview with W. Druml, December 13, 2021.

15 Lesky to Deutsch, March 25, 1966. Collections of the Medical University of Vienna (Josephinum), Lesky-Korrespondenzen 22 (D‑F, 1964–66), 78.

16 “Dumb Bruno,” as Lüdke was nicknamed in his German neighborhood, was charged with murder in 1943 after having committed several acts of theft and having been sterilized due to mental retardation. Lüdke proved easy to influence during manipulative questioning from ambitious detectives and despite contradictory evidence, was pinned with most of Germany’s unsolved murder cases—a number of 53, with an additional three attempted murders. After the police investigation, Lüdke was transferred to the Institute of Criminological Medicine in Vienna (*Kriminalmedizinisches Institut*), where he was examined and experimented on by members of the criminal(ist) academic elite. In what seems to have been the only such case at the institute, Lüdke was subjected to anthropological measurements and spinal punctures after highly-dosed alcohol intoxication. He also had, in a procedure that foreshadowed his death, a plaster cast face mask taken. (In a similar manner, face masks of Jewish internees in the Vienna Prater stadium were taken in 1939, shortly before they were transported to Buchenwald concentration camp; see [20]). Institute director Philipp Schneider handed in his resignation because he opposed using Lüdke to test aconitine-coated bullets. Ferdinand Schoen was discussed as his successor, a friend of the 1st Medical Clinic’s Josef Meller and, like Meller, Wilhelm Beiglböck, Hermann Möschl and Dietrich Roller, a member of the Luftwaffe and fervent Nazi. It is still unclear whether Lüdke ultimately met his demise due to poisoned bullets, or if he died of hypothermia or oxygenation experiments in the pressure chamber [21].

17 Collections of the Medical University of Vienna (Josephinum), AS 3524, Interview with B. Schneeweiß, July 28, 2020.

18 Michael Hubenstorf published Popper’s testimony in 1989 without naming the source or the alleged perpetrator [23, p. 260] and revealed both names to the authors in a personal communication on December 7, 2021. Ludwig Popper (1904–1984) had to flee the Nazis in 1938, which makes it unlikely he was a direct eyewitness.

19 The Diplomatic Archive Center of the Ministry of Foreign and European Affairs, La Courneuve, AUT 2590, Sécurité Militaire/Poste de Vienne, Renseignements sur Dr. Eppinger, June 25, 1946.

20 AT-OeStA, AdR, ZNsZ, GA Eppinger, Hans.

21 AT-OeStA, AdR, ZNsZ, GA Eppinger, Hans. “Alles andere als ein wirklicher Nationalsozialist”; “wird als äußerst minderwertiger Charakter beschrieben”; “Intrigant, Verleumder, brutal und rücksichtslos”; “man bezeichnet ihn als den schäbigsten Charakter der medizinischen Fakultät”.

22 UAW, Med. Dek., 442-1935/36, PA Eppinger, Hans.

23 Loosely translated: “whichever-ocrat” or “depending-on-ocrat.”.

24 AT-OeStA, AdR, ZNsZ, GA Eppinger, Hans. June, 1941, Sicherheitsdienst des Reichsführers-SS, Abschnitt Wien (Hauptsturmführer Hermann) an die NSDAP, Gauleitung Wien, Gaupersonalamt. “Eppinger’s suspected half-Jewish descent put him at risk of losing his job in Cologne. His grandmother was a Salomon of Prague and Eppinger had been the name of a classically ‘protected’ Jewish family in Austria.” Thaler states Eppinger’s descent from a “privileged Prague Jewish family.”

25 UAW, Med. Dek., 442-1935/36, PA Eppinger, Hans. The opening speech reached its climax with Eppinger’s quote “Wissen ist Macht, Macht aber nicht Wissen” (“Knowledge is power, but power isn’t knowledge”).

26 AT-OeStA, AdR, ZNsZ, GA Eppinger, Hans.

27 UAW, Med. S 11, Lehrkörper, Enthebungen 1937–1946; UAW, 1. Med. Klinik 108.

28 Collections of the Medical University of Vienna (Josephinum), AS 3524, Interview with B. Schneeweiß, July 28, 2020.

29 UAW, 1. Med. Klinik 104–105. e.g., recommendations for Hans Popper and Josef Faltitschek, both of which were expelled on March 12, 1938. See https://gedenkbuch.univie.ac.at/ [accessed on 30/11/2021]. Special thanks to Sabina Seehofer for assistance in navigating the extensive records.

30 AT-OeStA, AdR, UWFuK, BMU PA, Sign 21, Deutsch, Erwin.

31 City Archive Neustadt an der Weinstraße, Bestand PA, Nr. 190. Deutsch, Erwin. Eppinger and Dietlen were both members of the DGIM (German society for internal medicine) executive board, so it is possible that Eppinger facilitated the contact. See their entries at https://www.dgim-history.de/ [accessed November 30, 2021].

32 In addition to the bans from patient contact and from publishing, as well as the multiple coerced transfers. It was not until 1960 that the working hours were accredited.

33 Arolsen Archives, Sign. 6311020484, query regarding DEUTSCH ERWIN 12/04/1917, August 20, 1946.

34 UAW, I. Med. Klinik 24.

35 UAW, Med. Dek., 442-1935/36, PA Eppinger, Hans.

36 Collections of the Medical University of Vienna (Josephinum), AS 3524, Interview with B. Schneeweiß, July 28, 2020.

37 Pernkopf received a workspace at the Neurological institute to finish his work, for which he used execution victims of the Nazi judicial system.

38 The VdU (*Verband der Unabhängigen*) was absorbed in 1956 by the FPÖ (*Freiheitliche Partei Österreichs*), which still exists today.

39 Risak achieved rehabilitation four years later following a more merciful report by evaluators Fellinger and Hans Hoff.

40 Collections of the Medical University of Vienna (Josephinum), AS 3524, Interview with B. Schneeweiß, July 28, 2020.

41 According to Thaler, the student rhyme “Beim Herrn Professor Wenckebach sind nur die ersten Bänke wach” also applied to Lauda. (“Under Professor Wenckebach only the first rows are awake.”).

42 AT-OeStA, AdR, UWFuK, BMU PA, Sign 21, Deutsch, Erwin. Wien, November 12, 1958. “Referat über Dozent Dr. Erwin Deutsch, der in der Kommissionssitzung vom 22.01.1958 für die Verleihung des Titels eines a.o. Professors vorgeschlagen wurde.”

43 Such slides with edematous enlarged intercellular spaces could be the result of agonal or post-mortem tissue alterations or artefacts due to inadequate histological fixation.

44 Collections of the Medical University of Vienna (Josephinum), AS 3524, Interview mit B. Schneeweiß, July 28, 2020.

45 Collections of the Medical University of Vienna (Josephinum), AS 3524, Interview mit B. Schneeweiß, July 28, 2020.

46 AT-OeStA, AdR, UWFuK, BMU PA, Sign 21, Deutsch Erwin, Personenstandesblatt.

47 AT-OeStA, AdR, UWFuK, BMU PA, Sign 21, Deutsch Erwin; StA NW, Bestand PA Nr. 190, Deutsch, Erwin.

48 German Federal Archives (BArch), SSO, 146B, Nr. 1256–1257. Hubenstorf M. Biographisches Lexikon österreichischer Nazi-Ärzte (forthcoming). Special thanks to Michael Hubenstorf for sharing his extensive research.

49 Personal information from Michael Hubenstorf.

50 Collections of the Medical University of Vienna (Josephinum), AS 3524, Interview with B. Schneeweiß, July 28, 2020.

51 Most notably to Bern/Switzerland in 1949 (3 months at the Medical University Clinic under W. Frey), to the US in 1953/54 (7 months at the Dept. of Physiology and Pharmacology, Wayne University, Detroit/Michigan on a Fulbright scholarship) and in 1960 (1 month at the Department of Medicine, Washington Univ., St. Louis/Missouri with visits to Detroit, New York, Princeton) and to Oslo/Norway in 1961.

52 Collections of the Medical University of Vienna (Josephinum), AS 2646‑4, Erwin Deutsch 1964–1974, Der Ausbau der Klinik.

53 UAW, II. Med. Klinik, Besetzung (GZ: 94, 1973/74).

54 Shortly before being called to lead the 1st Medical Clinic in Vienna in 1964, Deutsch had applied for the analogous position in Innsbruck. He was listed *secundo loco*, and Braunsteiner *tertio loco*—the position nevertheless went to Braunsteiner. Universisty Archives Innsbruck, Akten der Medizinischen Fakultät, Reihe “Berufungsakten 1869–1969”, Akt “Medizinische Klinik/Innere Medizin 1962–1964” (Personal information from Peter Goller, head archivist in Innsbruck).

55 According to the conservative Austrian Cartellverband (ÖCV), Firnberg supported Braunsteiner, but Kreisky prevented Braunsteiner’s appointment. Hartmann, G. 2017. *em. Univ.-Prof. FA Dr. Dr. h. c. mult. Herbert Braunsteiner *[Online]. https://oecv.at/Biolex/Detail/12400117 [accessed April 4, 2022].

56 Braunsteiner also claimed in a letter to former dean Husslein that Deutsch had influenced the appointment process using information on these political implications. He also mentions that the 2nd Clinic’s assistants protested against Geyer’s appointment by way of sending a petition to the Ministry of Science and Research. UAW, II. Med. Klinik, Besetzung (GZ: 94, 1973/74). Braunsteiner to Husslein, 8 July 1976.
